# MRI-guided microwave ablation of hepatic malignancies: 4-year institutional outcomes

**DOI:** 10.1371/journal.pone.0322917

**Published:** 2026-10-26

**Authors:** Daniel Markus Düx, Marcel Gutberlet, Julian Glandorf, Simon Schröer, Kristina Imeen Ringe, Frank Wacker, Bennet Hensen, Lena Becker

**Affiliations:** 1 Diagnostic and Interventional Radiology, Hannover Medical School, Hannover, Germany; 2 STIMULATE-Solution Centre for Image Guided Local Therapies, Magdeburg, Germany; 3 Division of Interventional Radiology, Department of Radiology and Biomedical Imaging, Yale School of Medicine, New Haven, Connecticut, United States of America; Yonsei University College of Medicine, KOREA, REPUBLIC OF

## Abstract

**Purpose:**

We assessed the safety and efficacy of magnetic resonance (MR)-guided microwave ablation for treating hepatic malignancies.

**Methods:**

This retrospective single-center review evaluated patients who underwent MR-guided microwave ablation for hepatic malignancies between 02/15/2017–04/22/2020. Complications were classified following CIRSE guidelines. Local tumor progression (LTP), local tumor recurrence (LTR), non-target progression (NTP), progression-free survival (PFS) and time to progression (TTP) were assessed, with data presented as mean ± standard deviation.

**Results:**

Sixteen patients (60.75 ± 14.4 years; 9 males, 7 females) underwent 17 MR-guided microwave ablation for 21 hepatic malignancies (17 (81.0%) hepatocellular carcinoma, 1 (4.8%) cholangiocarcinoma, 2 (9.5%) metastases, and 1 (4.8%) suspicious lesion in a patient with primary sclerosing cholangitis). Lesion size was 12.2 ± 4.8 mm and total procedure time 138 ± 53 minutes. Complications included fever (N = 2), abdominal pain (N = 2), biloma (N = 1), and reactive cholecystitis (N = 1). These resolved with pain medication for one patient and antibiotics for another. No tumor recurrence was observed at ablation sites (LTP and LTR = 0%) at 36.7 ± 28.2 months. NTP occurred in 11 patients (68.8%) with a TTP of 14.8 ± 11.4 months. PFS was 44 months (95% CI: 10–85 months). Three patients died during follow-up — one from complications after liver transplantation and two from disease progression. Post-ablation liver transplantation was performed in two patients.

**Conclusion:**

MR-guided microwave ablation for hepatic malignancies is safe and effective, with no local tumor recurrences noted over a follow-up period of 36.7 ± 28.2 months.

**Clinical significance:**

MR-guided microwave ablation is a promising minimally invasive treatment for hepatic malignancies, demonstrating no LTR at 36.7 ± 28.2 months. Precise, radiation-free needle placement in real-time and effective targeting of small tumors may further improve future patient care.

## Introduction

Primary or secondary hepatic malignancies (HM) have the fastest-growing incidence and are the leading cause of cancer-related deaths [1,2]. Although surgery has shown 5-year survival rates of up to 70%, < 30% of patients qualify for resection or liver transplantation due to comorbidities, chronic liver disease, or advanced tumor stages [3–5]. Historically, percutaneous tumor ablation has evolved as promising minimally invasive treatment for HM in patients unsuitable for surgery [6], offering comparable tumor control, but improved morbidity, mortality, and reduced healthcare costs in patients with colorectal liver metastases and hepatocellular carcinoma (HCC) [7,8]. Consequently, ablation is increasingly recognized as a first-line treatment option. Techniques such as radiofrequency ablation (RFA), microwave ablation (MWA), cryoablation, and irreversible electroporation (IRE) have gained traction over the past years, all methods aiming for effective tumor tissue destruction while sparing healthy tissue [9–12]. MWA, in particular, offers advantages such as reduced heat sink effect, greater resilience to tissue changes (e.g., impedance, water content), higher intra-tumoral temperatures, and larger ablation volumes in shorter times [6,9,13,14]. Consequently, MWA has shown lower local recurrence rates compared to RFA [15], especially in larger tumors and those near large vessels, with recurrence rates < 2% for HCC ≤ 3 cm [16–20].

Image guidance is critical for safe and effective ablation. X-ray fluoroscopy, ultrasound (US), and computer tomography (CT) face challenges like radiation exposure, limited field of view (FOV), and poor visualization of the target or its proximity to sensitive surrounding anatomy as noted in the CIRSE guidelines [21]. Research is ongoing to improve imaging techniques and enhance fusion methods to compensate for technical limitations. Fusion is a promising and powerful tool for future intervention, but is still complex, time consuming, and needs specialized expertise. Current software is often limited in terms of compatibility with different imaging systems. Fusion can be unreliable in areas with organ movement like in thoracic and abdominal interventions due to registration issues [22].

Magnetic resonance imaging (MRI) provides a promising solution with real-time needle guidance, no ionizing radiation, excellent spatial and temporal resolution, and the potential to measure temperature within the liver during ablation in real-time [23]. MRI guidance is especially effective in minimizing collateral damage and offers valuable intra-procedural information, such as thermometry, crucial for therapy monitoring [23–28].

The value of MR-guided liver ablations has already been demonstrated [29]. However, most studies reported follow-up ≤ 3 years, and oncologic outcomes of MR-guided microwave ablation (MRgMWA) with long follow-up is scarce [29]. We aimed to evaluate the results of MRgMWA for HM over a follow-up period of ≥ 4 years.

## Methods

### Patients

This retrospective single-center cohort study included consecutive patients who underwent MRgMWA of HM between 02/15/2017–04/22/2020 (IRB-No.: 3227−2016). Written informed consent was obtained for all procedures. All cases underwent multidisciplinary discussion in our hepatobiliary tumor board. In our hepatobiliary tumor board, all cases were thoroughly discussed. MR guidance was reserved as a problem-solving modality for HM that could not be reliably visualized or safely targeted under ultrasound or CT – for example very small lesions, lesions in hazardous locations such as the hepatic dome adjacent to the diaphragm or heart, or lesions in which ablation under conventional image guidance had previously failed or was not feasible. The use of MR guidance was decided on an individual, indication-driven basis as part of the interdisciplinary tumor-board recommendation, and procedures were scheduled individually within an oncologically appropriate time frame, taking into account the limited availability of interventional MRI slots under general anesthesia. Because selection followed this individualized clinical pathway rather than a predefined screening process, and given the retrospective design, a formal screening cohort with sequential eligibility stages was not defined. Additional inclusion criteria included a sufficient coagulation status (thrombocytes > 50,000/µl, INR < 1.5), a maximum lesion diameter of ≤ 5 cm, and no more than three target lesions. Exclusion criteria included patients with non-MR-compatible implants or metal and those ineligible for anesthesia.

### Equipment

All procedures were performed in a 1.5 Tesla (T) MRI (Aera; Siemens Healthineers, Erlangen, Germany) with 70 cm bore-size. Ablation was conducted with an MRI-compatible, CE certified AveCure microwave system (MedWaves Inc., San Diego, CA) operating at 902–928 MHz, which was positioned next to the scanner’s bore. MR-compatible MWA-antennas of 14–16 Gauge with active tip lengths of 2–4 cm were chosen based on the target lesion’s size and location. These antennas had integrated temperature sensors, adjusting output power and generator frequency to maintain the desired temperature. The generator automatically adapted the output power and microwave frequency based on its thermometry feedback. A radiofrequency (RF)-shielded liquid crystal display monitor (Siemens Healthineers, Erlangen, Germany) was used for real-time MR-fluoroscopy. Sequences and imaging parameters for MR-guided ablation are summarized in Table 1.

**Table 1:** Sequences and imaging parameters for MR-guided ablation.

Sequence	TR (ms)	TE (ms)	Slice thickness (mm)	Matrix
**Planning**				
t1_vibe_dixon_ax	6.69	2.3	4	220 x 320
T2_tse_fs_ax	3993	102	5	384 x 512
ep2d_diff_b50_800_ax	8600	60	5	288 x 384
t1_vibe_fs_ax	3.34	1.2	4	192 x 254
**Antenna Placement**				
TrueFISP_cor	605	1.9	5	128 x 128
**Post-ablation Protocol**				
T2_tse_fs_ax	3993	102	5	384 x 512
ep2d_diff_b50_800_ax	8600	60	5	288 x 384
t1_vibe_fs_ax (with Dotarem)	3.3	1.2	4	192 x 254

Abbreviations: *ax:* axial*; cor: coronal; Vibe:* Volumetric Interpolated Breath-hold Examination*; DWI:* Diffusion-weighted imaging*; TSE:* Turbo Spin Echo*; TrueFISP:* Fast imaging with steady-state free precession*; (w)/o:* (with and) without; *fs:* Fat saturation; *ep2d:* Echo Planar 2D Imaging; *b50_800:* b-values 50–800.

For MR-fluoroscopy, a near-real-time, steady-state free precession fluoroscopic sequence (BEAT_IRTTT sequence) was employed, generating three parallel images with a frame rate of 330 ms per image. These images were angulated from the skin entry point of the antenna to the target.

### Patient treatment

Procedures were performed under general anesthesia by a radiologist with 5 years of experience in MRgMWA (B.H.). Pre-treatment imaging was acquired within six weeks prior to intervention.

The workflow can be subdivided into positioning/planning, fingertipping (defining the entry site of the antenna), puncture, ablation, and post-ablation control (Fig 1). The individual pictured in Fig 1 has provided written informed consent (as outlined in PLOS consent form) to publish their image.

**Fig 1:**
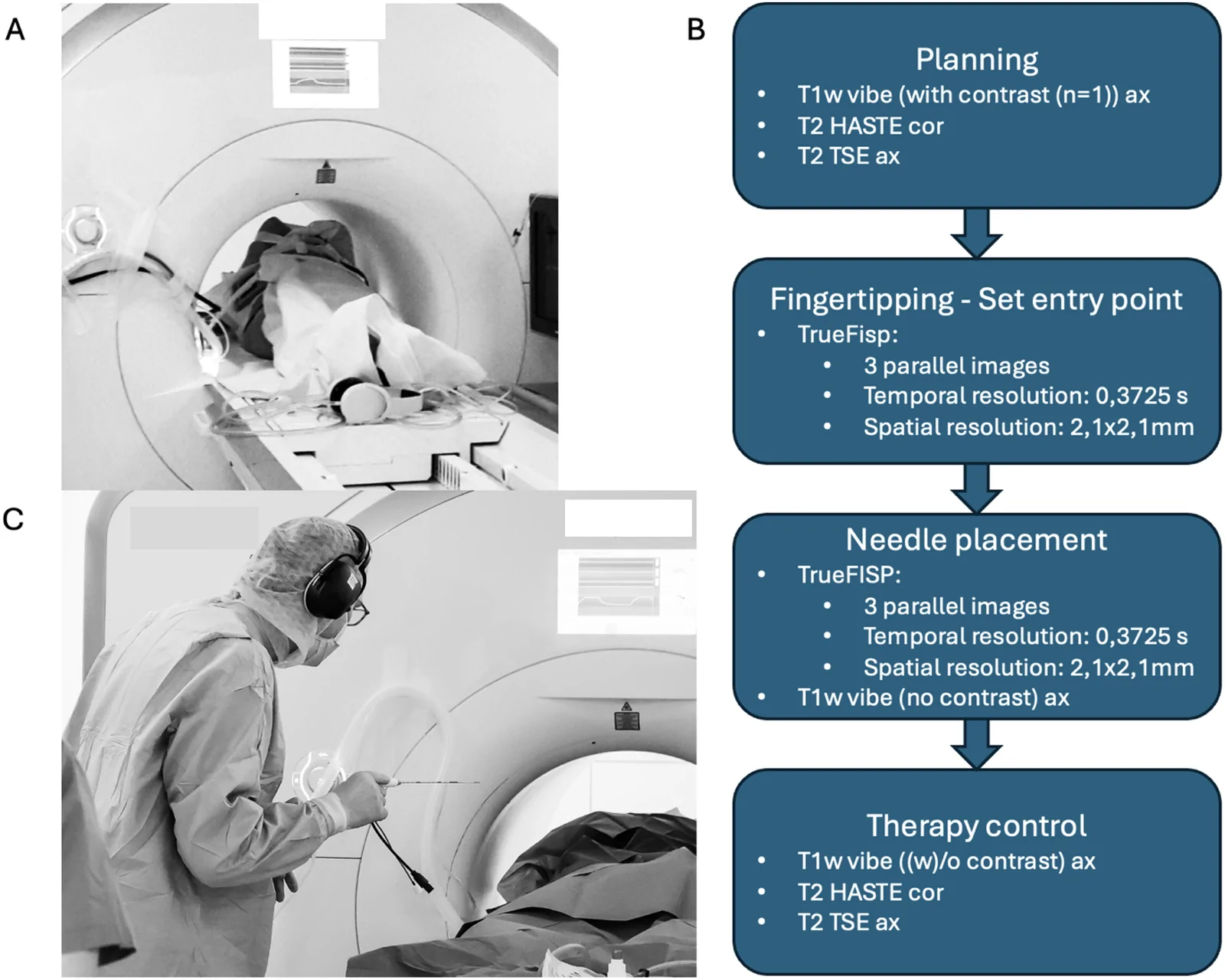
Workflow of MR-guided ablation of the liver. The procedure included positioning, planning, fingertipping (set entry site), needle placement including the ablation and therapy control. A: Shows patient positioning for lateral needle access under general anesthesia in supine position. The patient’s right arm was placed anteriorly. The patient has already been moved into the MRI for scanning. B: Flow-chart of each step of the intervention. Listed are all sequences used in each phase. C: Interventionalist holding the antenna for microwave ablation right before needle placement. Abbreviations: w: weighted; ax: axial; Vibe: Volumetric Interpolated Breath-hold Examination; DWI: Diffusion-weighted imaging; TSE: Turbo Spin Echo; TrueFISP: Fast imaging with steady-state free precession; (w)/o: (with and) without.

Lesion targeting was primarily accomplished using T2-weighted and non-contrast T1-weighted sequences. In one case, where unenhanced images provided limited demarcation, contrast was used. Fingertipping (Fig 2) and puncturing (Fig 3) with the MWA-antenna was done using MR-fluoroscopy; accurate MWA-antenna placement was verified with a T1w sequence. Post-procedural imaging consisted of T2w and contrast-enhanced T1w sequences (Fig 3).

**Fig 2:**
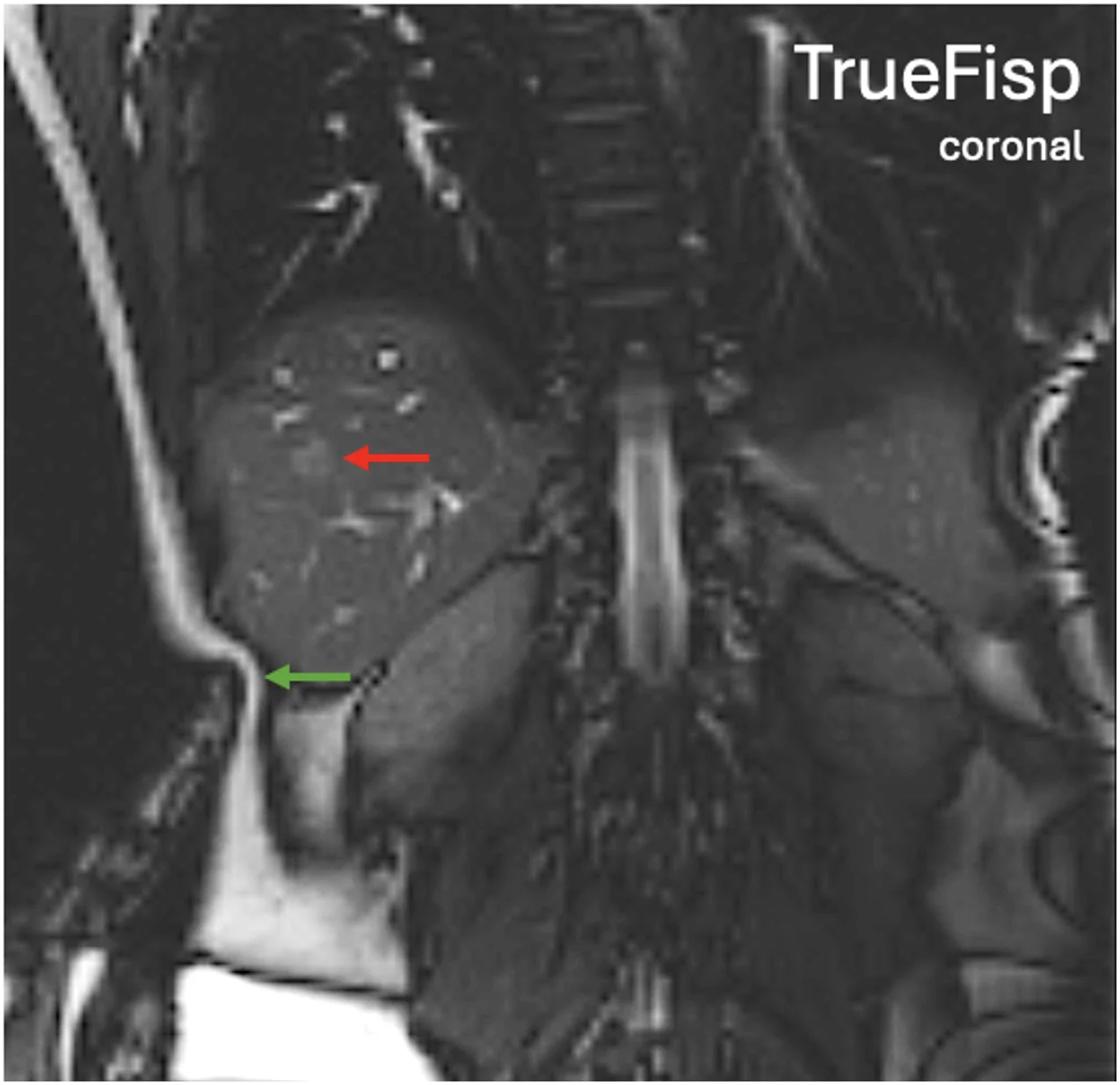
Fingertipping technique: locating needle entry point. Coronal image from a TrueFISP sequence illustrating the fingertipping method for identification of the entry point. Following pathway planning, the interventionalist aligned his finger (green arrow) with the slice where the lesion was located. The hyperintense area marking the hepatic metastasis in liver segment 8 is highlighted by the red arrow in this illustration. Once the entry point was determined, the skin area was prepared for puncturing with the microwave antenna, including disinfection, draping and local anesthesia of the liver capsule. Abbreviations: TrueFISP: Fast imaging with steady-state free precession.

**Fig 3:**
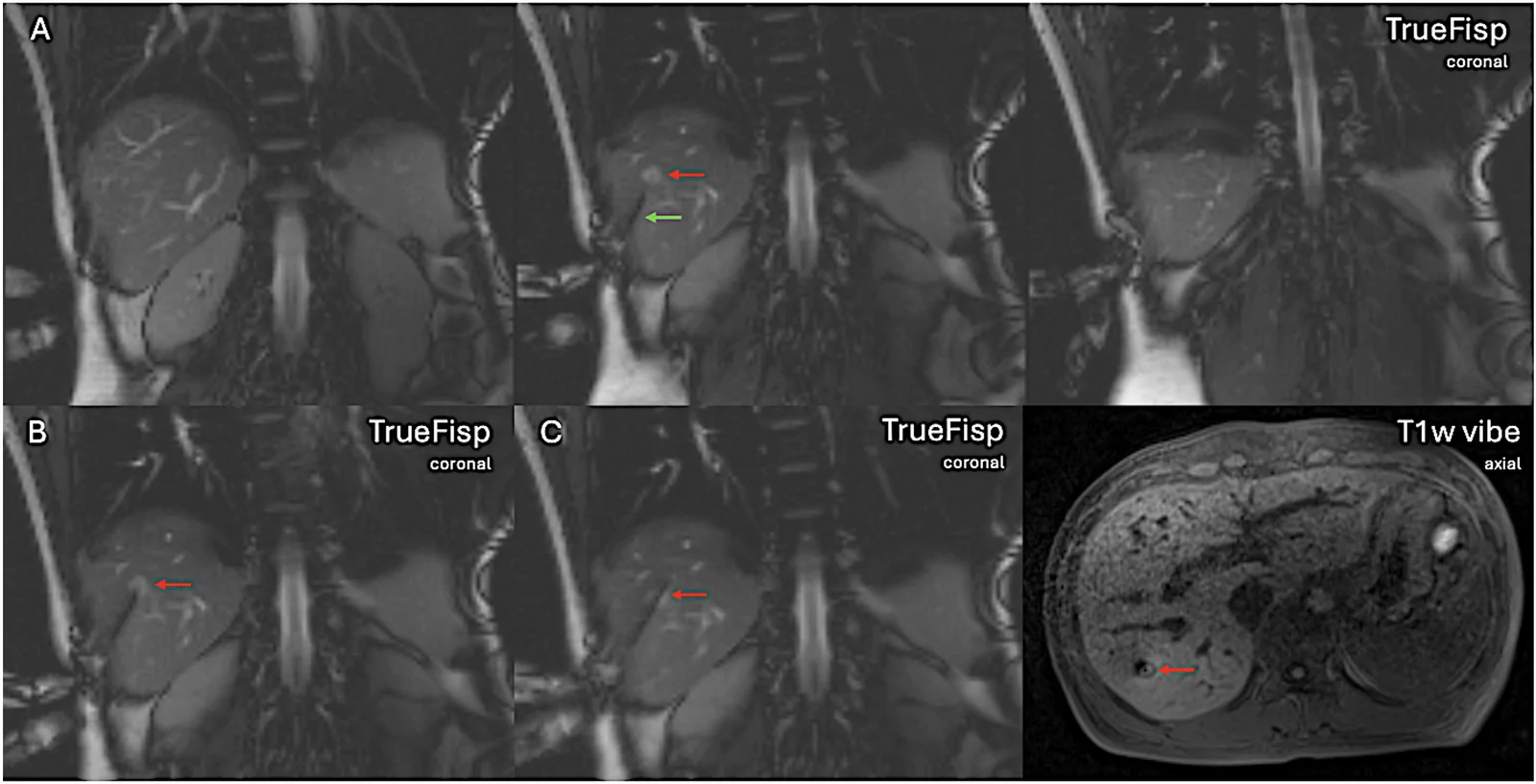
Placement of the microwave antenna. MR Fluoroscopy was utilized to position the microwave antenna for ablation on a hepatic metastasis in liver segment 8. A: A set of three parallel coronal images in the TrueFISP sequence revealed a hyperintense lesion (red arrow), with the interventionalist aligning the needle to the middle slice (antenna: green arrow). B + C: Continuing advancement of the needle towards central placement within the lesion in real-time. D: Needle position within the metastasis prior to ablation. The noise in this image was caused by electromagnetic interference from the MWA generator. Abbreviations: TrueFISP: fast imaging with steady-state free precession; TSE: Turbo Spin Echo.

### Data analysis

Patient demographics and technical details were documented. Technical success was defined as achieving complete ablation zone overlap on immediate post-ablation imaging, with a target safety margin of 0.5 cm. Tumors adjacent to the liver capsule or vessels were considered fully ablated if this safety margin was achieved elsewhere, as illustrated in Fig 4. Complications were reported according to the Cardiovascular and Interventional Radiological Society of Europe (CIRSE) classification system [30].

**Fig 4:**
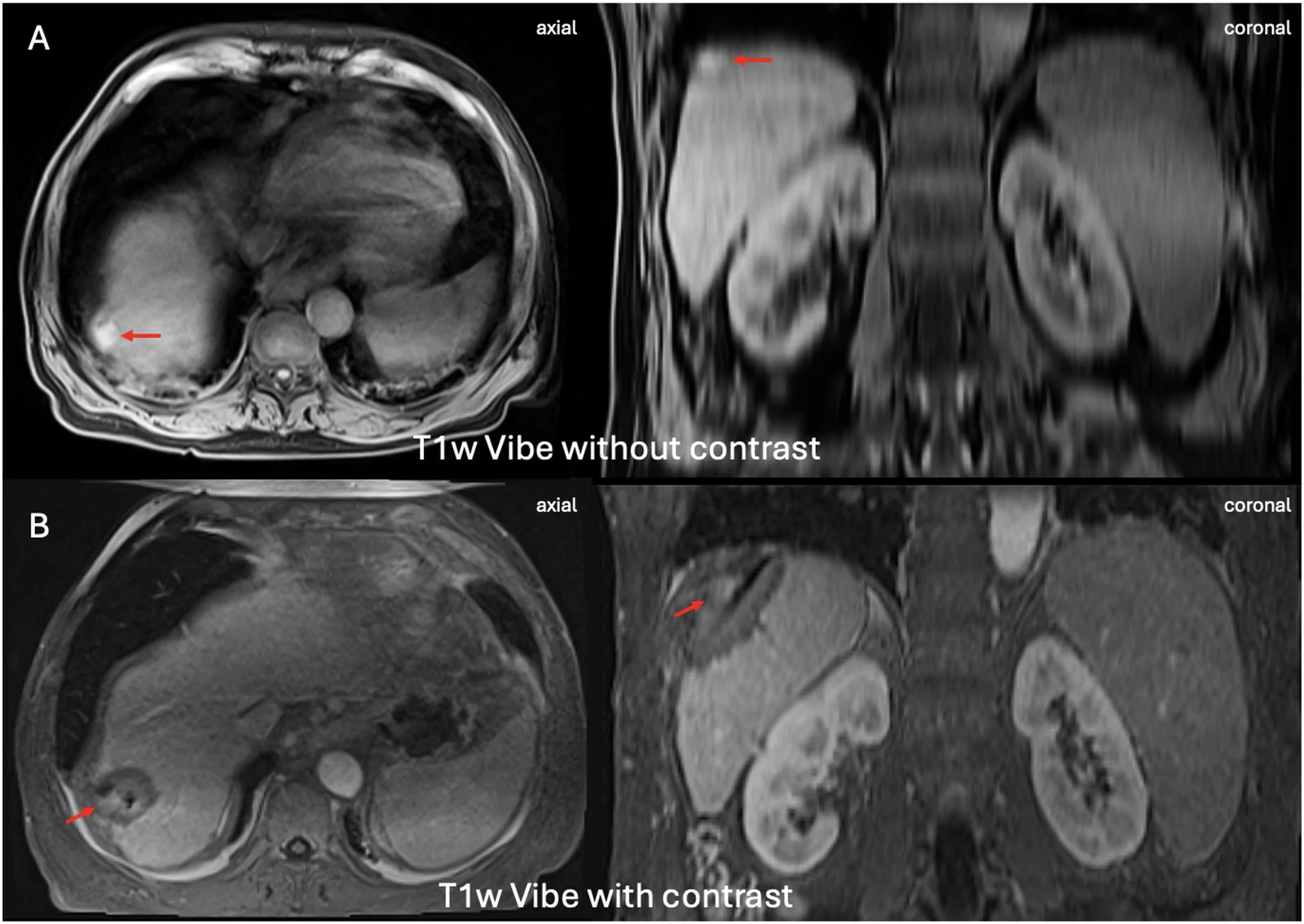
Pre- and post-ablation images. Imaging of a patient with a history of HCC. Shown are pre- (A) and post-ablation (B) imaging. Tumor board ruled in favor of image-guided ablation of this singular lesion in liver segment 7. A: T1w Vibe sequences without contrast were used to identify a 2 cm lesion in liver segment 7. B: T1w Vibe sequences with contrast were employed to ensure complete ablation with a 5 mm safety margin without any acute complications. Abbreviations: HCC: Hepatocellular carcinoma; TSE: Turbo Spin Echo; w: weighted; Vibe: Volumetric Interpolated Breath-hold Examination.

### Follow-up

Follow-up imaging with contrast-enhanced CT or MRI was performed six to eight weeks post-ablation and every four months during the first year, with extended intervals with further extension based on progression-free survival. Patients were seen by the referring department, which included additional tests such as tumor markers. Retrospective analysis was based on each patient’s latest available follow-up. Local tumor progression (LTP) and local tumor recurrence (LTR) were defined as residual or recurrent HM within or immediately adjacent to the ablation zone [31]. Non-target progression (NTP) included new or growing tumor, which was not treated with MRgMWA. Progression-free survival (PFS) was defined as the time from the MRgMWA to disease progression or death from any cause. Time to progression (TTP) was reported descriptively as the mean time from MRgMWA to documented progression, restricted to patients who experienced a progression event; TTP was not analyzed as a competing-risk endpoint and is provided to complement PFS by characterizing how rapidly progression occurred in those patients in whom it did occur.

### Statistical analysis

Statistical analyses were performed using commercially available software (SPSS, version 28.0.1.1 (14); IBM, Chicago IL, Illinois). Data is presented as mean ± standard deviation (SD). Kaplan‑Meier analysis was used to estimate the median PFS, with corresponding 95% confidence intervals reported.

During the preparation of this manuscript, the authors used Claude Opus 4.5 (Anthropic) in order to improve readability and language of the manuscript. After using this tool, the authors reviewed and edited the content as needed and take full responsibility for the content of the publication.

## Results

16 patients (average age 60.75 ± 14.4 years; 9 males, 7 females) underwent 17 MRgMWA treating 21 HM, including primary liver cancer (N = 18; 85.7%), metastases (N = 2; 9.5%), and a suspicious lesion for HCC in a patient with primary sclerosing cholangitis (N = 1; 4.8%). Patient demographic and tumor characteristics are summarized in Table 2.

**Table 2:** Patient demographic information and tumor characteristics.

Patient (N = 16)	
Age [years]	60.8 ± 14.4
BMI [kg/m^2^]	25.8 ± 3.6
Gender	
Female (N; %)	7 (43.8%)
Male (N; %)	9 (56.3%)
Hepatic Malignancies (N = 21)	
Primary liver tumor	
HCC (N; %)	17 (81.0%)
CCC (N; %)	1 (4.8%)
Lesion of unknown origin (suspicious of HCC) (N; %)	1 (4.8%)
Hepatic metastasis	
Breast cancer (N; %)	1 (4.8%)
Colon cancer (N; %)	1 (4.8%)
Liver segments	
I	0 (0.0%)
II	1 (4.8%)
III	1 (4.8%)
IVa	2 (9.5%)
IVb	0 (0.0%)
V	1 (4.8%)
VI	0 (0.0%)
VII	8 (38.1%)
VIII	8 (38.1%)
Mean lesion size [mm]	12.2 ± 4.8
Tumorstatus (N = 16)	
T1 (N; %)	3 (18.8%)
T2 (N; %)	10 (62.5%)
T3 (N; %)	2 (12.5%)
T4 (N; %)	1 (6.3%)
N0 (N; %)	15 (93.8%)
N3 (N; %)	1 (6.3%) (breast cancer)
M0 (N; %)	13 (81.3%)
M1 (N; %)	3 (18.8%)
Liver cirrhosis (N = 12; 75.0%)	
Etiology	
HCV (N; %)	7 (43.8%)
HBV (N; %)	1 (6.3%)
Ethyl-toxic (N; %)	2 (12.5%)
Unknown origin (N; %)	2 (12.5%)
Child-Pugh classification (Liver cirrhosis patients only, N = 12)	
A (N; %)	7 (58.3%)
B (N; %)	5 (41.7%)
BCLC classification (HCC patients only, N = 12)	
A (N; %)	11 (91.7%)
B (N; %)	1 (8.3%)
Laboratory results (N = 16)	
Liver function parameters	
ALT (U/l; N ± SD)	47 ± 39 (N = 4 elevated)
AST (U/l; N ± SD)	42 ± 18 (N = 9 elevated)
CHE (kU/l; N ± SD)	6 ± 2 (N = 1 decreased)
Cholestatic parameters	
γ-GT (U/l; N ± SD)	60 ± 28 (N = 11 elevated)
Bilirubin (µmol/l; N ± SD)	21 ± 16 (N = 4 elevated)
Coagulation parameters	
Quick (%; N ± SD)	89 ± 20 (N = 2 decreased)
INR (N ± SD)	1.1 ± 0.1 (N = 3 elevated)
Tumor marker	
AFP (ng/ml; N ± SD)	43 ± 107 (N = 2 elevated)

Abbreviations: *HCC* Hepatocellular carcinoma*; CCC* cholangiocarcinoma; *N°* number.

^a^Continuous data is presented as means ± standard deviation; categorical data is presented as the counts/sample (percentage).

^b^TNM stage was assigned according to the entity-specific UICC/AJCC 8th-edition classification for the respective primary tumor; the single N3 entry refers to the breast-cancer primary in the patient with breast-cancer liver metastasis.

^c^BCLC staging is reported only for the patients with HCC (N = 12).

^d^Child-Pugh classification applies only to the patients with liver cirrhosis (N = 12); the 4 non-cirrhotic patients (2 metastases, 1 CCC, 1 PSC-associated lesion) were not classified according to Child-Pugh.

All interventions were technically successful, and patients were discharged after an average hospitalization period of 3.6 ± 1.5 days. Technical information and complications are provided in Table 3.

**Table 3:** Technical information and complications.

Technical data (N ± SD)	
Mean energy [Joule]	17.7 ± 4.6 kJ
Mean ablation time [min]	11.1 ± 1.9 min
Mean procedure time [min]	138 ± 53 min
Complications (N; %)	
Fever	2 (11.8%): Grade 1, Grade 3 (required antibiotics)
Abdominal pain	2 (11.8%): Grade 1, Grade 3 (required pain medication)
Biloma	1 (5.9%): Grade 1
Reactive cholecystitis	1 (5.9%): Grade 1

Abbreviations: *kJ:* Kilo Joule*; min:* Minutes; *N°:* number.

^a^Continuous data is presented as means ± standard deviation; categorical data is presented as the counts/sample (percentage).

The mean lesion size was 12.2 ± 4.8 mm (range: 6–23 mm). Repositioning of the MWA antenna was required in nine treatments to ensure complete coverage, with an average of 2.2 applicator positions. The desired ablation temperature of 120 °C resulted in a mean ablation time of 11.1 ± 1.9 minutes and an average energy deposition of 17.7 ± 4.6 kJ. Total procedure time was 138 ± 53 min. Complications included fever (N = 2; 11.8%), abdominal pain (N = 2; 11.8%), biloma (N = 1; 5.9%), and reactive cholecystitis (N = 1; 5.9%). As per the CIRSE guidelines, three (17.6%) grade 1 and two (11.8%) grade 3 complications were noted. All complications resolved, two after appropriate treatment.

The mean follow-up period was 36.7 ± 28.2 months. No evidence of residual or recurrent viable tumor within ablation zones was detected on follow‑up imaging up to the last available follow‑up, indicating a LTP and LTR rate of 0%. NTP was identified in 11 (68.8%) patients (10 patients with primary liver cancer and 1 patient with metastasis) at an average TTP of 14.8 ± 11.4 months post-ablation. To assess the robustness of the local-control findings, we additionally performed a sensitivity analysis excluding the patient with the histologically unconfirmed PSC-associated lesion; LTP and LTR remained at 0% (15 patients, 16 ablations), while NTP (11/15 [73.3%]), median PFS (44 months) and mean TTP (14.8 ± 11.4 months) changed only marginally, supporting the robustness of the primary findings.

Kaplan‑Meier estimated a median PFS of 44 months (95% CI: 10–85 months). PFS at six months, one, two, three and four years were 86.7%, 73.3%, 53.3%, 53.3%, 33.3%, respectively (Fig 5). Among these patients, one developed a seeding metastasis in soft tissue, possibly due to inadequate tract ablation. Further management was administered based on tumor board consensus. Three patients died at 2, 7, and 48 months post-ablation, with one death related to surgery post-liver transplantation, and two due to disease progression. Two (12.5%) patients underwent liver transplantation at 2 months post-procedure, one remained tumor-free at 48 months post-ablation.

**Fig 5:**
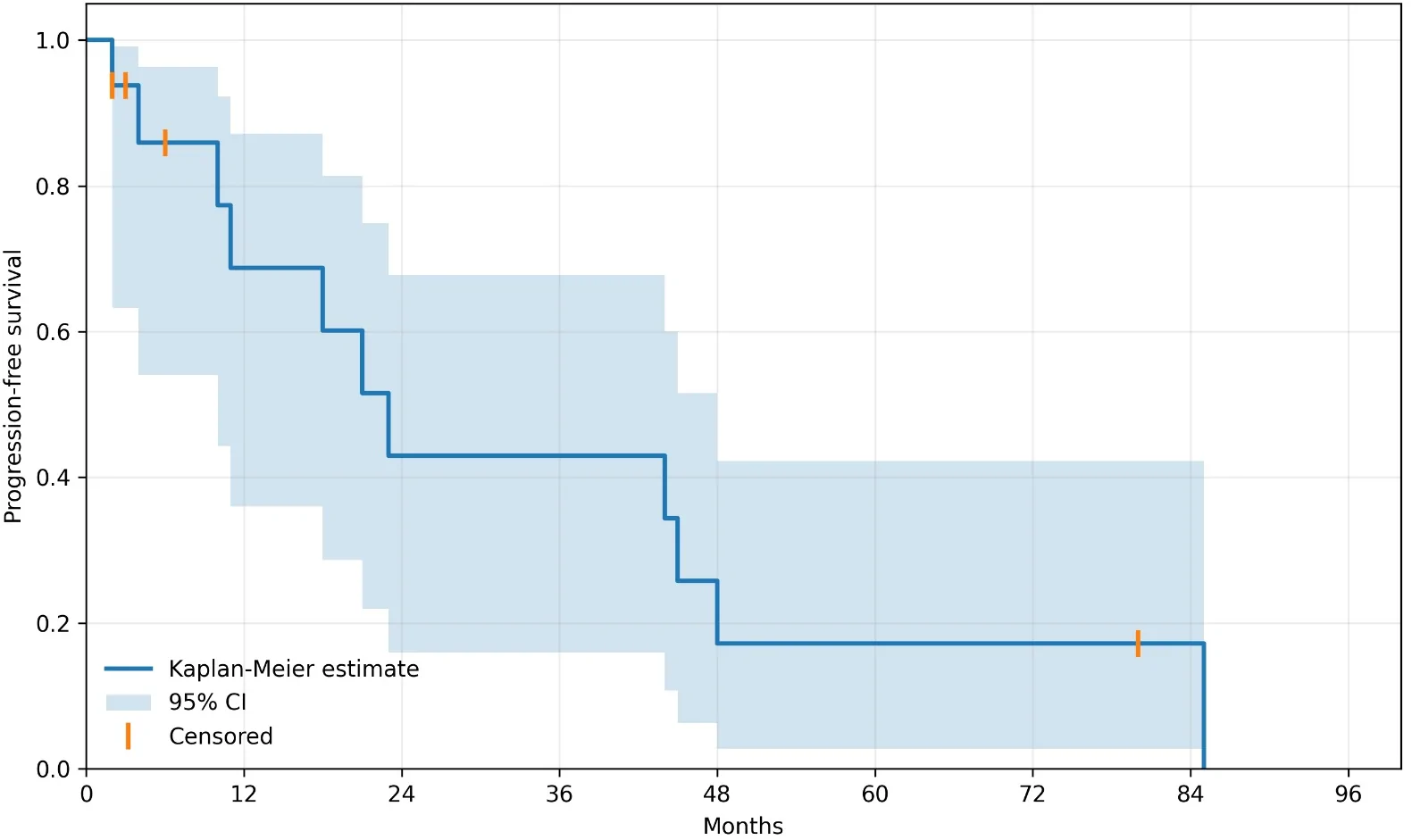
Kaplan-Meier analysis of tumor progression after MRgMWA. Kaplan-Meier analysis estimated the median PFS with 44 months (95% CI: 10 - 85 months) for all patients incorporating the latest follow-up data for each patient. This graph illustrates the proportion of patients remaining progression-free over time. The numbers at risk (patients alive and not censored) at 0, 12, 24, 36, 48, 60, 72, 84, and 96 months were 16, 8, 5, 5, 3, 2, 2, 1, and 0, respectively.

## Discussion

Several studies have demonstrated promising results regarding safety and efficacy using MR-guided liver ablation [29]. However, these studies are highly heterogenous, utilizing different ablation techniques (e.g., RFA, MWA, laser ablation, and cryoablation), different MRI magnet types (open or closed-bore) and field strength (ranging from 0.2 T to 1.5 T). These factors can affect patient selection, visualization and consequently outcomes. Imaging quality, for instance, may limit patient selection to larger tumors. Indeed, most studies report HM sizes ranging from 1.54 ± 0.77 cm [11] to 4.7 ± 1.8 cm [32] for ablation. However, larger tumor size is associated with higher recurrence rates after ablation, affecting the oncologic outcome [33]. Groeschl et al. studied 450 patients undergoing MWA for 875 hepatic masses and found that patients with tumors ≥ 3 cm had poorer recurrence-free survival (hazard ratio: 1.60, 95% CI: 1.02–2.50, p = 0.039). In our study the mean lesion size measured 12.2 ± 4.8 mm (range: 6–23 mm), treated in a 1.5 T closed-bore MRI. No evidence of LTR or LTP during a mean follow-up of 36.7 ± 28.2 months occurred supporting this observation.

Weiss et al. is the only other study that evaluated small HM < 1.2 cm in 45 patients using MRgMWA, with a mean size of 0.9 ± 0.21 cm [34]. At a follow-up period of 24.7 ± 14.0 months no LTP was observed matching our results. However, only 12 (28%) patients had a NTP at 21 ± 6 months. In our cohort NTP was identified in 11 (68.8%) patients at an average TTP of 14.8 ± 11.4 months post-ablation. The differences in NTP and TTP may be due to different patient selection criteria. In our cohort, 81.0% (n = 17) patients had HCC, which is known as a more recurrent tumor entity after ablation probably due to underlying liver disease [33]. Weiss et al. reported 25.5% (n = 11) patients with HCC.

The cohort comprises tumor entities treated with both curative and palliative intent. Differences in treatment intent, concomitant systemic therapy, and tumor-specific surveillance schedules are potential confounders of the pooled PFS, TTP, and NTP estimates and should be considered when interpreting these time-to-event outcomes. Endpoints reflecting the local technical success of MRgMWA (LTP and LTR) are less susceptible to this confounding than systemic progression endpoints and can be considered as treatment success.

To our knowledge, one prospective study exists with 47 patients [35]. A total of 13 (26%) complications occurred (10 minor (20%); 3 major (6%)), which is similar to our cohort. Median overall survival was 41.6 (IQR: 26.4) months. Four (8%) patients had LTP, but these tumors with LTP were significantly larger with 3.0 ± 1.0 cm compared to 1.5 ± 0.6 cm (p = 0.006), which supports that oncologic outcomes are excellent in MRgMWA of small HM. NTP was observed on 30 (64%) patients after a median time of 13.8 (IQR 2.3) months.

In the past, ablation of HM has primarily been incorporated into clinical guidelines for the treatment of unresectable HM [33,36], and surgery has traditionally been considered the only curative treatment option. However, continued research in small, resectable HM is needed, especially in context of the previously published COLLISION trial [7]. Patients with colorectal cancer liver metastasis (≤ 3 cm) were enrolled. 148 patients were randomly assigned to ablation and 148 patients to resection. The trial was terminated before the study ended due a substantially reduced mortality rate (surgery 2.1% (n = 3) and MWA 0% (n = 0)). No significant difference in overall survival (HR 1.05; 95% CI, 0.69–1.58; p = 0.83) was seen at 28.8 months, with a conditional probability of 90.5% in proving the non-inferiority hypothesis. No significant differences were observed in local and distant progression-free survival. However, ablation showed advantages in terms of the total number of adverse events (19% versus 46%; p < 0.0001), serious adverse events (7% versus 20%; p < 0.004), length of hospital stay (1 day versus 4 days, p < 0.0001), and local control (HR 0.184; 95% CI, 0.040–0.838; p = 0.029). Consequently, balancing morbidity and mortality relative to similar oncologic outcomes ablation should be favored over surgery in patients with colorectal liver metastases.

However, the optimal management of small HM is still debated, arguing that the individual tumor characteristics, location, liver function, and the risk of complications must be considered when choosing between surgery and ablation [37]. Indeed, small HM can be poorly visualized on CT or ultrasound and ablation is then performed by orienting at anatomical landmarks. MRgMWA offers excellent visibility for most tumors, even without contrast, thus allowing targeted, tissue sparing ablation. Moreover, MR-imaging can be freely adjusted to match the needle angulation, making it straightforward to target tumors in challenging locations.

Our study faced limitations associated with a small sample size and its retrospective nature, leading to inconsistent follow-up. All procedures were performed by a single interventional radiologist with five years of dedicated MRgMWA experience. While this ensured a high level of procedural consistency and minimized operator-related variability within the study, it also limits the external validity and generalizability of our results, particularly with respect to the learning curve, technical success rate, and complication profile observed at centers with multiple operators or less MRgMWA experience. It is important to note that there was no pre-selection for a specific tumor entity. Management of various tumor entities varies and depends on factors such as growth rate and size. Future research should concentrate on investigating MRgMWA in a single tumor entity; however, large cohorts are currently scarce due to the limited number of centers offering MRgMWA. One reason behind this is the limited availability of clinically approved MWA devices, some of which have only recently received approval. The first MWA with CE mark for MRgMWA was the Medwaves system used in this study. This system is less effective in heat deposition compared to the systems used for CT or US. Additionally, the lack of insulation and cooling in the MWA antenna may often lead to an elongated ablation zone, resulting in inefficient workflows that may require repositioning and may limit potential needle trajectories. Newer MR-compatible MWA systems, such as the ECO system (ECO-200, ECO Medical Instrument Co., China), have insulated and cooled MWA antennas, which might offer more efficiency. Future devices with improved performance will further improve the efficacy of this treatment. In the future, multicenter studies with larger patient cohorts should be performed to confirm the value of MR-guidance for liver ablation presented here.

## Conclusion

MRgMWA for HM is advancing in the field of interventional oncology and serves as a valuable, innovative, minimally invasive tool to improve patient outcomes. Particularly, the precise ablation of small HM, which are difficult to visualize with CT or US guidance, is a key strength of MR guidance, as it allows to target the HM directly. In carefully selected patients, MRI-guided ablation of small HM may offer favorable local control, with added benefits such as tissue preservation and quicker recovery. However, given the limited cohort size, these preliminary findings require confirmation in larger studies before outcomes can be compared with surgery. These advantages may enhance future patient management by reducing recovery time and minimizing complications.

### Main points

1. MR-guided microwave ablation (MRgMWA) of hepatic malignancies (HM) is safe.
2. MRgMWA achieved 100% technical success in small HM (12.2 ± 4.8 mm).
3. No local tumor recurrences occurred at 36.7 ± 28.2 months.
4. Non-target progression occurred in 11 (68.8%) patients at 14.8 ± 11.4 months.

## Abbreviations

CCC: Cholangiocarcinoma
CIRSE: Cardiovascular and Interventional Radiological Society of Europe
CT: Computer tomography
FDG PET: Fluorine-18-Fluorodeoxyglucose Positron Emission Tomography
G: Gauge
FOV: Field of View
HCC: Hepatocellular carcinoma
HM: Hepatic malignancy
LTP: Local tumor progression
LTR: Local tumor recurrence
Min: Minutes
MWA: Microwave ablation
MRI: Magnetic resonance imaging
MRgMWA: MR-guided microwave ablation
NTP: Non-target progression
PRFS: Proton resonance frequency shift
RF: Radiofrequency
RFA: Radiofrequency ablation
SD: Standard deviation
US: Ultrasound
W: weighted
T: Tesla
TTP: Time to progression
